# Relationship of In Vitro Toxicity of Technetium-99m to Subcellular Localisation and Absorbed Dose

**DOI:** 10.3390/ijms222413466

**Published:** 2021-12-15

**Authors:** Ines M. Costa, Noor Siksek, Alessia Volpe, Francis Man, Katarzyna M. Osytek, Elise Verger, Giuseppe Schettino, Gilbert O. Fruhwirth, Samantha Y. A. Terry

**Affiliations:** 1 Department of Imaging Chemistry and Biology, School of Biomedical Engineering and Imaging Sciences, King’s College London, London SE1 7EH, UK; ines.costa@kcl.ac.uk (I.M.C.); noorlsiksek@gmail.com (N.S.); francis.man@kcl.ac.uk (F.M.); katarzyna.osytek@kcl.ac.uk (K.M.O.); elise.verger44@gmail.com (E.V.); 2 Memorial Sloan Kettering Cancer Center, Molecular Imaging Group, Department of Radiology, New York, NY 10065, USA; volpea1@mskcc.org; 3 National Physical Laboratory, Department of Medical Radiation Sciences, Teddington TW11 0LW, UK; giuseppe.schettino@npl.co.uk; 4 Faculty of Engineering and Physical Sciences, University of Surrey, Guilford GU2 7XH, UK; 5 Comprehensive Cancer Centre, Imaging Therapies and Cancer Group, School of Cancer and Pharmaceutical Sciences, King’s College London, London SE1 1UL, UK; gilbert.fruhwirth@kcl.ac.uk

**Keywords:** auger electron therapy, dosimetry, molecular radionuclide therapy, radiobiology, sodium iodide symporter, technetium, triple-negative breast cancer cells

## Abstract

Auger electron-emitters increasingly attract attention as potential radionuclides for molecular radionuclide therapy in oncology. The radionuclide technetium-99m is widely used for imaging; however, its potential as a therapeutic radionuclide has not yet been fully assessed. We used MDA-MB-231 breast cancer cells engineered to express the human sodium iodide symporter-green fluorescent protein fusion reporter (hNIS-GFP; MDA-MB-231.hNIS-GFP) as a model for controlled cellular radionuclide uptake. Uptake, efflux, and subcellular location of the NIS radiotracer [^99m^Tc]TcO_4_^−^ were characterised to calculate the nuclear-absorbed dose using Medical Internal Radiation Dose formalism. Radiotoxicity was determined using clonogenic and γ-H2AX assays. The daughter radionuclide technetium-99 or external beam irradiation therapy (EBRT) served as controls. [^99m^Tc]TcO_4_^−^ in vivo biodistribution in MDA-MB-231.hNIS-GFP tumour-bearing mice was determined by imaging and complemented by ex vivo tissue radioactivity analysis. [^99m^Tc]TcO_4_^−^ resulted in substantial DNA damage and reduction in the survival fraction (SF) following 24 h incubation in hNIS-expressing cells only. We found that 24,430 decays/cell (30 mBq/cell) were required to achieve SF_0.37_ (95%-confidence interval = [SF_0.31_; SF_0.43_]). Different approaches for determining the subcellular localisation of [^99m^Tc]TcO_4_^−^ led to SF_0.37_ nuclear-absorbed doses ranging from 0.33 to 11.7 Gy. In comparison, EBRT of MDA-MB-231.hNIS-GFP cells resulted in an SF_0.37_ of 2.59 Gy. In vivo retention of [^99m^Tc]TcO_4_^−^ after 24 h remained high at 28.0% ± 4.5% of the administered activity/gram tissue in MDA-MB-231.hNIS-GFP tumours. [^99m^Tc]TcO_4_^−^ caused DNA damage and reduced clonogenicity in this model, but only when the radioisotope was taken up into the cells. This data guides the safe use of technetium-99m during imaging and potential future therapeutic applications.

## 1. Introduction

Auger electron (AE) therapy is an attractive option to treat disseminated cancers [1]. AEs have a very short range (<1 µm) with linear energy transfer (LET) reaching 26 keV/µm, setting them apart from α- and β-particles [1,2]. Consequently, AE-emitters induce cell toxicity when in close proximity to radiosensitive structures (i.e., nuclear DNA). Targeting AE-emitters into cancer cells can therefore specifically kill cancer cells while theoretically minimising radiation toxicity to surrounding healthy tissues [1]. Currently, [^123^I]I-MAPI is the most promising AE-radiopharmaceutical undergoing clinical translation for the treatment of glioblastoma multiforme [3].

Technetium-99m has a half-life of 6.02 h, wide availability, and emits γ-rays (140 keV) ideal for single-photon emission computed tomography (SPECT) [4]. Importantly, it also decays by internal conversion, resulting in the average emission of 1.1 internal conversion electrons and 4.4 AEs per decay [1], thus potentially being a theragnostic tool in AE-therapy. Previous studies showed technetium-99m to damage DNA, reduce cell survival, and slow tumour growth [5,6,7].

Technetium-99m in the chemical form of [^99m^Tc]TcO_4_^−^ is specifically taken up by cells expressing the human sodium/iodide symporter (hNIS), which is a transmembrane glycoprotein that normally mediates iodide uptake into thyroid follicular cells [8]. hNIS presents with endogenous expression restricted to the thyroid, gastric mucosa, salivary, and lacrimal glands [8]. Its anion transport selectivity is low, and [^99m^Tc]TcO_4_^−^ and other imaging radiotracers are also transported. hNIS has been exploited for imaging, [^131^I]I^−^-therapy and as a reporter gene [9]. hNIS has also been used for controlled uptake of [^99m^Tc]TcO_4_^−^ to determine its radiotoxicity [10,11]. However, a radiobiology-informed characterisation of technetium-99m effects to predict the efficacy of potential technetium-99m-based AE-therapy and to assess its radiobiological risk when used as an imaging agent remains elusive [12,13].

Here, we used breast cancer cells with defined hNIS expression as a model of controlled [^99m^Tc]TcO_4_^−^ uptake to determine cellular technetium-99m radiotoxicity and nuclear-absorbed dose and to provide reference data in comparison to external beam radiation therapy (EBRT).

## 2. Results

### 2.1. In Vitro Uptake and Efflux of [^99m^Tc]TcO_4_^−^

**Figure 1:**
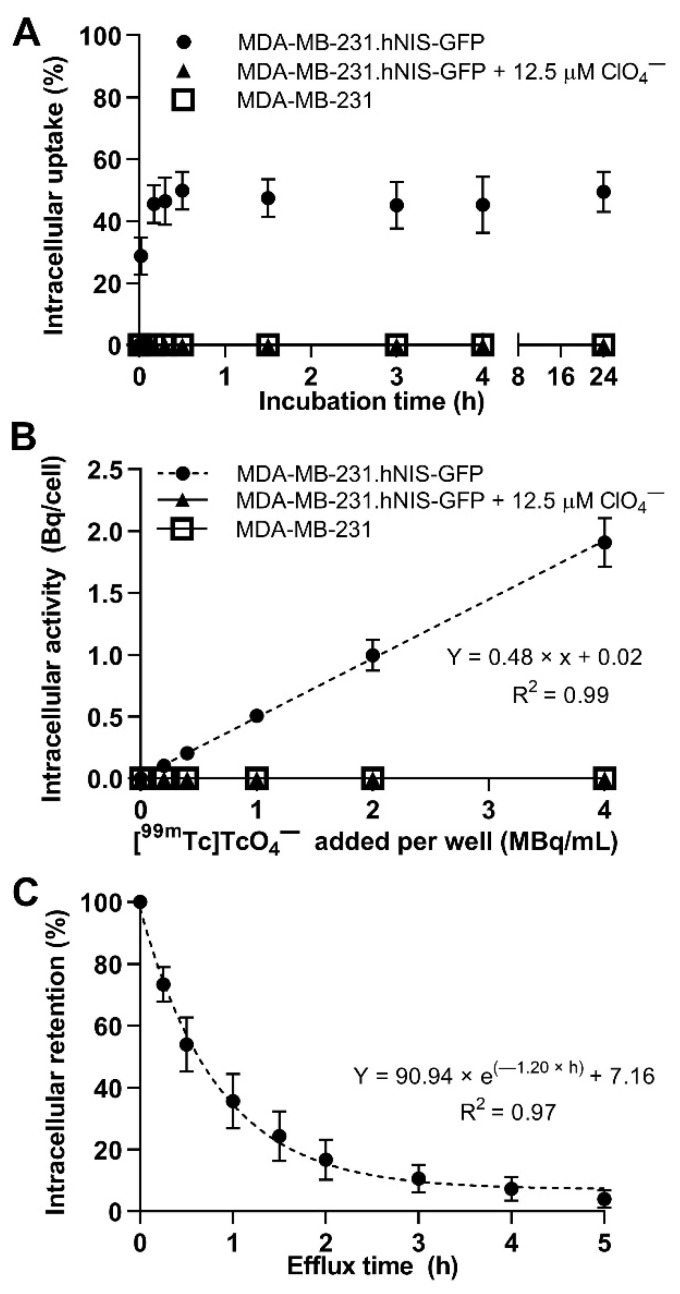
Uptake studies in MDA-MB-231.hNIS-GFP. (**A**) Intracellular uptake of 0.2 MBq/mL [^99m^Tc]TcO_4_^−^ over time and (**B**) intracellular radioactivity after 0.5 h incubation with [^99m^Tc]TcO_4_^−^ with up to 4 MBq/mL in the presence and absence of ClO_4_^−^. Dashed line represents linear regression fit. (**C**) Intracellular retention of [^99m^Tc]TcO_4_^−^ over time after an initial incubation of 0.5 h with a mono-exponential fit (dashed line).

We engineered MDA-MB-231 cells to stably express hNIS-green fluorescent protein fusion reporter (hNIS-GFP) [9], FACS-sorted them, determined hNIS-GFP expression stability and subcellular localisation (Figure S1), and quantified [^99m^Tc]TcO_4_^−^ uptake into these cells (uptake reached a plateau of 46.5% ± 7.6% after 0.5 h; Figure 1A). Controls included parental MDA-MB-231 cells and radiotracer uptake competition with ClO_4_^−^ in MDA-MB-231.hNIS-GFP cells, both showing negligible uptake (Figure 1A,B). Increasing radiotracer concentrations resulted in a linear increase of uptake, reaching 1900 ± 200 mBq/cell in our studies (Figure 1B). This was expected for the used concentrations (0–208 pM), which were much lower than the reported Michaelis-Menten constants for hNIS-fluorescent protein fusion reporters (i.e., ~10 µM [14]). Moreover, radiotracer retention analyses revealed a mono-exponential efflux with a retention half-life of 0.58 h (95% confidence interval (CI) = [0.47; 0.72]; Figure 1C).

### 2.2. Technetium-99m and EBRT-Induced DNA Damage

**Figure 2:**
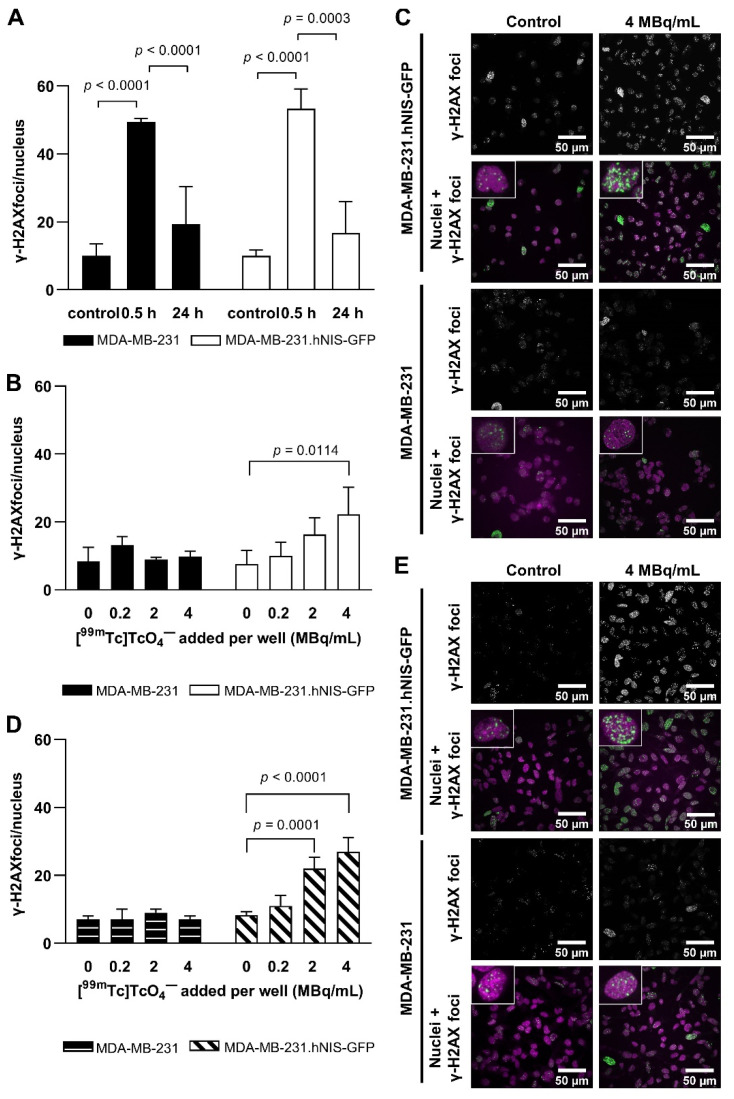
DNA damage. (**A**) Average number of γ-H2AX foci per nucleus for untreated and irradiated (0.5 h and 24 h incubation post-2 Gy EBRT) or (**B**) cells incubated with [^99m^Tc]TcO_4_^−^ for 0.5 h with up to 4 MBq/mL. (**C**) Representative confocal images of cells indicating γ-H2AX foci (green) within nuclei (magenta). (**D**,**E**) Data from analogous experiments as in (**B**,**C**) but for 24 h of incubation with [^99m^Tc]TcO_4_^−^.

Average numbers of γ-H2AX foci per nucleus significantly increased 0.5 h after EBRT with 2 Gy irrespective of the cell type compared to unirradiated cells (Figure 2A). These decreased 24 h post-irradiation with no significant difference compared to control non-irradiated cells at 24 h (Figure 2A). No significant difference in radiosensitivity between parental and hNIS-GFP-expressing cells was observed (*p* = 0.9191).

When exposing cells to indicated [^99m^Tc]TcO_4_^−^ concentrations for 0.5 h, MDA-MB-231.hNIS-GFP cells showed significantly more DNA damage (22.3 ± 7.9) compared to either untreated MDA-MB-231.hNIS-GFP (7.6 ± 4.0) or [^99m^Tc]TcO_4_^−^-incubated MDA-MB-231 cells (9.7 ± 1.6) (2-way ANOVA: *p* < 0.0196; Figure 2B,C). The effects on DNA damage were dependent on radiotracer concentration (0.5 h: *p* = 0.0370, 24 h: *p* < 0.0001; Figure 2B,D). An increase of incubation time to 24 h did not result in a significant increase of DNA damage (*p* = 0.1136; Figure 2D,E). Following 24 h recovery after 24 h radiotracer exposure, DNA damage was reduced significantly to background levels (Figure S2A,B), except for MDA-MB-231.hNIS-GFP cells treated with 4 MBq/mL, (16.1 ± 1.8 vs. 6.4 ± 1.3 foci/nucleus for untreated cells; *p* = 0.0256). No significant increase in DNA damage compared to untreated cells was observed in MDA-MB-231 cells with increasing concentrations of [^99m^Tc]TcO_4_^−^ at 0.5 h and 24 h (*p* = 0.1878 and *p* = 0.4411; Figure 2B,D). Notably, incubation of MDA-MB-231.hNIS-GFP cells with the decay product [^99^Tc]TcO_4_^−^ did not significantly increase DNA damage (*p* = 0.2500) (Figure S2C).

### 2.3. Cytotoxicity

**Figure 3:**
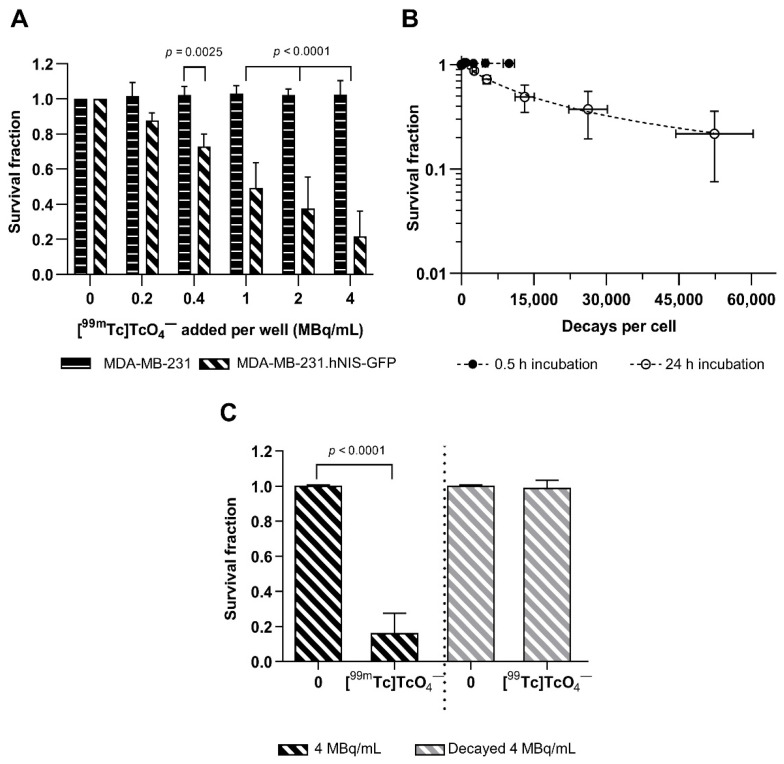
Cytotoxicity. (**A**) Survival fraction (SF) following 24 h incubation with [^99m^Tc]TcO_4_^−^. (**B**) SF of MDA-MB-231.hNIS-GFP cells as a function of cumulative intracellular decays per cell. Dashed lines represent least-square regression fitting. (**C**) SF of MDA-MB-231.hNIS-GFP cells following 24 h incubation with 4 MBq/mL [^99m^Tc]TcO_4_^−^ and decayed [^99^Tc]TcO_4_^−^. Statistical significance determined by Wilcoxon signed-rank *t*-test.

Incubation of MDA-MB-231.hNIS-GFP cells with [^99m^Tc]TcO_4_^−^ for 24 h decreased the survival fraction (SF) compared to untreated MDA-MB-231.hNIS-GFP cells or MDA-MB-231 cells treated with [^99m^Tc]TcO_4_^−^ (2-way ANOVA: *p* < 0.0001) (Figure 3A). We calculated that 1.87 MBq/mL (equivalent to 30 mBq/cell at 24 h taking into account technetium-99m decay and cell growth) and 24,430 decays/cell were required to reduce the SF of MDA-MB-231.hNIS-GFP cells to 0.37 (SF_0.37_) (95%CI = [SF_0.31_; SF_0.43_] Figure 3A,B). MDA-MB-231.hNIS-GFP cells incubated with [^99m^Tc]TcO_4_^−^ for only 0.5 h showed no clonogenic differences compared to untreated or [^99m^Tc]TcO_4_^−^ treated MDA-MB-231 cells (2-way ANOVA: *p* = 0.9873; Figure S3). The SF of MDA-MB-231 cells treated with [^99m^Tc]TcO_4_^−^ was not significantly different from that of untreated cells at 0.5 h and 24 h (*p* = 0.7379 and *p* = 0.7015, respectively; Figure 3A and Figure S3). There was also no SF reduction in MDA-MB-231.hNIS-GFP treated with decay product [^99^Tc]TcO_4_^−^ (Figure 3C).

### 2.4. Dosimetry

**Figure 4:**
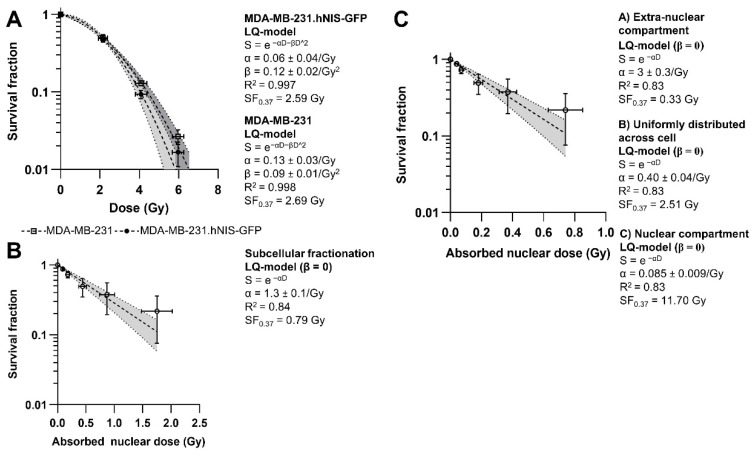
Dose-response curves of (**A**) MDA-MB-231 and MDA-MB-231.hNIS-GFP cells following EBRT. Survival Fraction (SF) of MDA-MB-231.hNIS-GFP cells treated with increasing concentrations of [^99m^Tc]TcO_4_^−^ for 24 h as a function of the nuclear-absorbed dose using (**B**) the subcellular fractionation method and (**C**) the volumetric method. SF was fitted to the linear-quadratic (LQ)-model (with β = 0) (dashed lines) and 95% confidence intervals (shaded areas).

When exposed to EBRT, the SF of MDA-MB-231.hNIS-GFP and MDA-MB-231 cells significantly decreased with increasing absorbed dose, with no significant difference between the two cell lines (*p* = 0.2045) (Figure 4A). Calculation of dosimetry for molecular radiotracers fundamentally requires knowledge of the subcellular distribution of the radioisotope. Classical cell fractionation methodology showed that only 4.7% ± 3.5% of [^99m^Tc]TcO_4_^−^ was in the nuclei. In contrast, volume-based calculations (see Supplementary Methods), assuming that [^99m^Tc]TcO_4_^−^ was uniformly distributed across the whole cell, estimated the nuclear [^99m^Tc]TcO_4_^−^ percentage to be 19.2% ± 8.3%. Using this data as input enabled the calculation of dose-response curves for MDA-MB-231.hNIS-GFP cells treated for 24 h with [^99m^Tc]TcO_4_^−^, wherein data was fitted using the linear-quadratic (LQ)-model with the constraint β = 0 (Figure 4B,C). Using the subcellular fractionation-derived value as input, an estimated absorbed dose to the nucleus of 0.79 Gy was delivered to reach SF_0.37_ (95%CI = [SF_0.29_;SF_0.45_]; Figure 4B). In contrast, relying on the volumetric approach, we estimated the delivered nuclear-absorbed doses to obtain SF_0.37_ depending on the assumed location of [^99m^Tc]TcO_4_^−^ within the cells, as shown in Figure 4C.

### 2.5. In Vivo Imaging and Tissue Distribution

**Figure 5:**
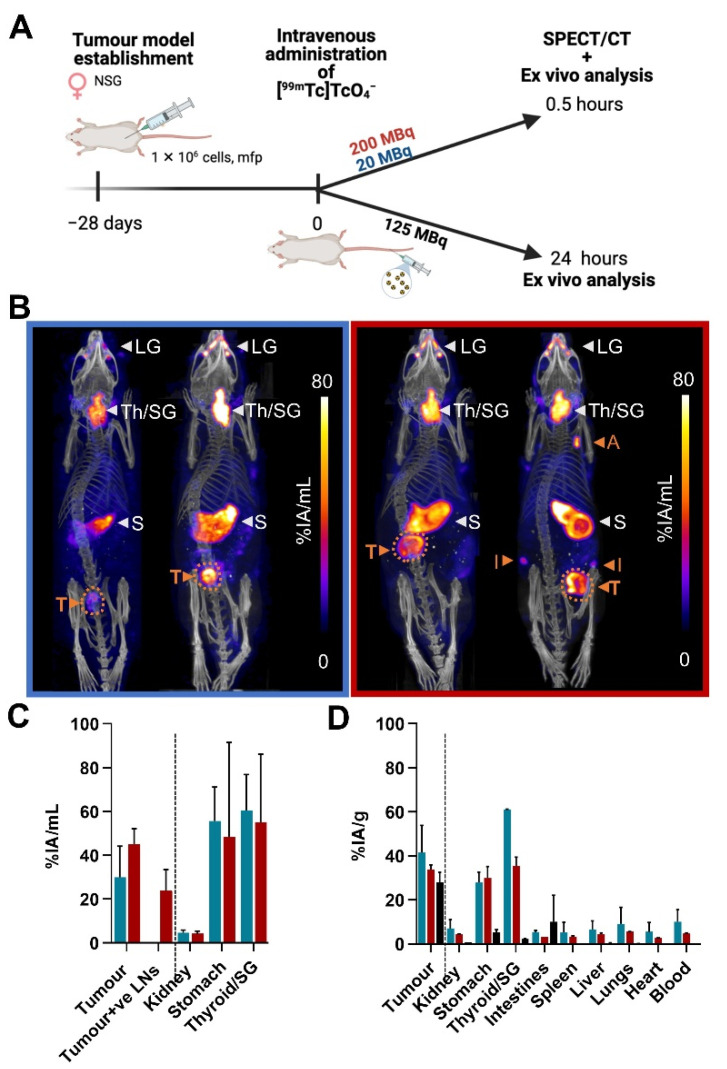
Biodistribution of [^99m^Tc]TcO_4_^−^ in MDA-MB-231.hNIS-GFP tumours. (**A**) Experimental scheme. (**B**) SPECT/CT imaging at 0.5 h post-administration of 20 MBq (left animals; blue) and 200 MBq (right animals; red) [^99m^Tc]TcO_4_^−^. CT images (grayscale) were overlayed with SPECT maximum intensity projections (MIP; hue). MIPs show the retention of [^99m^Tc]TcO_4_^−^ in endogenously NIS-expressing organs (white inscriptions: LG-lacrimal glands; S-stomach and Th/SG-thyroid/salivary glands) and MDA-MB-231.hNIS-GFP tumours and cancer-positive lymph nodes (orange inscriptions: A-Auxiliary lymph node; I-Inguinal lymph node; T-Tumour). (**C**) Quantitative radioactivity analysis from SPECT/CT images in (**B**). (**D**) Ex vivo analysis of harvested tissues. *N* = 2/cohort.

SPECT/Computed Tomography (SPECT/CT) images showed that [^99m^Tc]TcO_4_^−^ was taken up into endogenously hNIS-expressing organs (i.e., thyroid, stomach, and lacrimal glands; Figure 5A–C) as well as into primary MDA-MB-231.hNIS-GFP tumours (30% ± 14% percentage injected activity per mL (%IA/mL) and 45% ± 7% IA/mL for when 20 and 200 MBq were administered, respectively) and lymph nodes bearing metastases (23.8% ± 9.6% IA/mL). Based on SPECT/CT images and assuming that primary tumours were composed of compact spherical cells with a radius of 11 ± 2 μm, it was estimated that 21 ± 10 mBq/cell and 390 ± 60 mBq/cell were delivered to tumours following intravenous injection of 20 and 200 MBq, respectively. Moreover, 24 h post-administration of 125 MBq [^99m^Tc]TcO_4_^−^, the radiotracer was still retained within cancerous tissues (28.0% ± 4.5% percentage injected activity per gram (%IA/g) as well as in endogenous NIS-expressing organs (Figure 5D).

## 3. Discussion

Only few in vitro and in vivo radiotoxicity studies have been performed with technetium-99m so far [6,10,11,15,16,17,18,19], and the correlation of technetium-99m-induced radiotoxicity with intracellular activity remained elusive. Here, using an hNIS expression-afforded model for controlled [^99m^Tc]TcO_4_^−^ uptake into cells, we found that technetium-99m induced DNA damage and reduced cell survival manifested only when the radioisotope was taken up into cells (Figure 2B–E and Figure 3A). Notably, the technetium-99m decay product, the β-emitter technetium-99, did not exert any DNA damage under these conditions, further demonstrating the specificity of the observed effects to technetium-99m. Importantly, observed differences in DNA damage induction between hNIS-GFP-expressing and parental MDA-MB-231 cells were not due to hNIS-mediated changes in radiosensitivity as demonstrated by both cell lines suffering equal DNA damage when exposed to EBRT (Figure 2A).

While we reached up to 1900 ± 200 mBq/cell after 0.5 h incubation with [^99m^Tc]TcO_4_^−^, a longer incubation time of 24 h was needed to observe radiotoxicity (Figure 1B and Figure 3A). Therefore, a cellular concentration of 30 mBq/cell technetium-99m was required to reduce survival to 37%. This was about two-fold less than what was previously reported for rat thyroid follicular cells [11], whereby possible expression and uptake rate differences between hNIS-GFP in our cells and rat NIS in rat thyroid follicular cells, as well as differences in radiosensitivity were not accounted for. To better compare studies not performed under identical conditions, we estimated decays/cell, and in our work, 24,430 decays/cell were required to achieve SF_0.37_ (95% CI = [SF_0.31_; SF_0.43_]). This is substantially more than was required to achieve a 90% reduction of cell survival when using indium-111 and gallium-67 (3240 and 3600 decays/cell, respectively, over 60 min incubation) or thallium-201 (1000–1600 decays/cell over 90 min incubation) [20,21]. While this data stem from different cells, the discrepancies in toxicity effectiveness are likely to be caused by the lower emission of average AE energy released per decay for technetium-99m compared to indium-111, gallium-67, and thallium-201 (0.9 vs. 6.6, 6.9, and 20.9 keV, respectively [1]). Technetium-99m also presents with a larger ratio between total emitted photon energy and electrons per decay compared to other AE-emitters such as platinum-195m (7.78 vs. 0.42 [22]), which increases its likelihood of inducing undesirable radiation effects on normal tissues. Moreover, the short half-life of technetium-99m (6.02 h) is sub-optimal for AE-therapy [22,23]. Despite these disadvantages compared with traditional AE-emitters, technetium-99m remains a contender for theranostic applications as it is widely available, has a long-lived (half-life = 2.1 × 10^5^ years) daughter radionuclide, hence less likelihood of inducing undesired harm if detached from a targeting radiopharmaceutical, and is ideally suited for SPECT imaging [4].

The correlation of radiobiological effects with the absorbed dose in EBRT is well-understood and crucial to assess radiobiological risks and plan radiotherapy. The dose-response curves acquired here help to better understand the differences between EBRT and potential AE-therapy with technetium-99m in terms of biological response at the same absorbed radiation dose. Although the LQ-model is adequate for low LET-radiation such as in EBRT [12,24], we showed here that the dose-response curve of internal irradiation with [^99m^Tc]TcO_4_^−^ followed an LQ-model with a short sub-lethal damage repair half-life (high α/β), i.e., negligible quadratic term in the LQ-model (constrain β = 0) (Figure 4B,C). This difference in the survival curves (β = 0 vs. β > 0) may have been due to heterogeneous dose delivery, protracted exposure and lower dose rate of [^99m^Tc]TcO_4_^−^ over the 24 h incubation period (<1 Gy/h) when compared to a homogeneous high acute dose rate of 5 Gy/min in EBRT, thereby enabling cells to repair sub-lethal damage [24]. Moreover, the non-significant changes in DNA damage with increasing incubation time indicated a protracted exposure to [^99m^Tc]TcO_4_^−^. In addition, while DNA damage induced by EBRT was significantly repaired to background levels 24 h post-irradiation, DNA damage induced by [^99m^Tc]TcO_4_^−^ was not totally repaired compared to untreated cells for the largest concentration used (Figure S2A). Hence, our data suggest that lethal damage (α) is the key contribution for technetium-99m radiotoxicity.

For the determination of nuclear-absorbed doses, it was important to estimate the relative amounts of technetium-99m in the nucleus compared to the whole cell volume. Traditionally this was approached by subcellular fractionation. The soluble small molecule nature of [^99m^Tc]TcO_4_^−^ might, however, impact the accuracy of this approach [13]; hence, we also performed microscopy-aided cell volume determinations. First, using subcellular fractionation, a lower nuclear absorbed dose of 0.79 Gy compared to 2.59 Gy with EBRT was estimated to achieve SF_0.37_ (95%CI = [SF_0.29_; SF_0.45_] and [SF_0.35_; SF_0.39_], respectively; Figure 4A,B). Second, using microscopy-aided whole-cell and nucleus volume determinations, we calculated the nuclear-absorbed doses under the following different assumptions: that [^99m^Tc]TcO_4_^−^ was either (i) excluded from the nucleus, (ii) uniformly distributed across the whole cell, or (iii) located only in the nucleus (Figure 4C). Obtained values for the delivered nuclear-absorbed dose were (i) 0.33 Gy, (ii) 2.51 Gy, and (iii) 11.7 Gy, in line with the expectation of a dependency on relative technetium-99m proximity to the nucleus, i.e., the further away from the nucleus, the lower the estimated delivered nuclear-absorbed dose due to the short-range (<1 µm) energy deposition of AEs. Except for the unrealistic borderline case that all technetium-99m would be present in the nucleus, the obtained absorbed dose values were lower than for EBRT. These results were similar to a previous study in rat follicular cells that reported 1.2 ± 0.1 Gy and 2.6 ± 0.3 Gy for incubation with [^99m^Tc]TcO_4_^−^ (24 h) and irradiation with X-rays, respectively [11]. Notably, there were limitations stemming from a lack of accurate methods to determine precisely the relative distribution of [^99m^Tc]TcO_4_^−^ within cells and limitations in the Medical Internal Radiation Dose formalism (MIRD) formalism (i.e., assumption of uniform radioactivity distribution within cells, spherical shape of cells). Because of these limitations, we did not attempt to compare the relative biological effectiveness between AE-therapy with technetium-99m as modelled here and EBRT. However, the values obtained for nuclear-absorbed doses, the primarily lethal damage α component, and the long-term induced DSBs, suggest enhanced radiobiological effectiveness of technetium-99m compared to EBRT if technetium-99m was internalised into cells.

We also performed a non-exhaustive in vivo study to quantify the relative uptake of [^99m^Tc]TcO_4_^−^ (Figure 5). Our observations were in line with previously reported data on [^99m^Tc]TcO_4_^−^ uptake in organs [9,25]. Notably, our data showed that the retention of [^99m^Tc]TcO_4_^−^ in tumours was longer than expected from in vitro radiotracer efflux experiments and that [^99m^Tc]TcO_4_^−^ was still retained in the tumours 24 h after administration, i.e., the time required for [^99m^Tc]TcO_4_^−^ to induce radiotoxicity in our in vitro experiments. In our estimation, 0.5 h after administration of 20 MBq and 200 MBq [^99m^Tc]TcO_4_^−^ cellular activities of 21 ± 10 mBq/cell and 390 ± 60 mBq/cell were reached in the primary tumours. Therefore, at high administered [^99m^Tc]TcO_4_^−^ amounts, it may be possible to reach similar cellular radioactivity values in vivo compared to our in vitro experiments (Figure 1B) that showed substantial radiotoxicity (Figure 3A).

## 4. Materials and Methods

### 4.1. Cells

MDA-MB-231.hNIS-GFP were previously generated [9] with hNIS-GFP long-term expression and sub-cellular localisation analyses described here (Supplementary Methods). Cells were maintained in Dulbecco’s Modified Eagle Medium (1 g/L glucose) supplemented with 10% foetal bovine serum, 2 mM L-glutamine and 100 U/mL penicillin and 100 µg/mL streptomycin in a humidified 5% (v/v) CO_2_ atmosphere at 37 °C.

### 4.2. Radionuclides and EBRT

Technetium-99m was generator-eluted as Na[^99m^Tc]TcO_4_ (Guy’s and St Thomas’ NHS foundation Trust Radiopharmacy, London, UK) and used within one half-life of elution. EBRT was delivered using a gamma-irradiator calibrated using Gafchromic EBT3 films and performed at 5 Gy/min (Figure S4). To obtain the β-emitting daughter radionuclide technetium-99, solutions of 4 MBq/mL [^99m^Tc]TcO_4_^−^ were stored for ten half-lives and used at the same molecular concentrations as [^99m^Tc]TcO_4_^−^.

### 4.3. Cellular [^99m^Tc]TcO_4_^−^ Uptake and Efflux

Uptake: 2 × 10^5^ cells/24-well were incubated with 250 µL 0.2 MBq/mL [^99m^Tc]TcO_4_^−^ for up to 24 h or indicated concentrations (0–4 MBq/mL) for 0.5 h. Uptake specificity was demonstrated through competition with the co-substrate ClO_4_^−^ (12.5 µM, 0.5 h) before [^99m^Tc]TcO_4_^−^ addition. Following incubation, the medium was collected, cells were washed twice in phosphate buffered saline (PBS), and lysed in 1 M NaOH. Sample radioactivity was quantified with a gamma-counter (LKB-Wallack CompuGamma 1282, Turku, Finland) and results expressed as intracellular uptake percentage and intracellular radioactivity (Equations (1) and (2)).

(1) $$\mathrm{Intracellular}\;\mathrm{uptake}\;\left(\%\right)=\frac{\mathrm{Counts}\;\mathrm{per}\;\mathrm{minute}\;{\left(\mathrm{CPM}\right)}_{\mathrm{cells}\;}-{\;\mathrm{CPM}}_{\mathrm{background}}}{\left({\mathrm{CPM}}_{\mathrm{supernatant}}-{\mathrm{CPM}}_{\mathrm{background}}\right)+\left({\mathrm{CPM}}_{\mathrm{washes}}-{\mathrm{CPM}}_{\mathrm{background}}\right)+\left({\mathrm{CPM}}_{\mathrm{cells}}-{\mathrm{CPM}}_{\mathrm{background}}\right)}\times 100$$

(2) $$\mathrm{Intracellular}\;\mathrm{radioactivity}\;\left(\mathrm{mBq}/\mathrm{cell}\right)=\frac{\mathrm{Intracellular}\;\mathrm{uptake}\left(\%\right)\times \;\mathrm{activity}\;\mathrm{added}\;\mathrm{per}\;\mathrm{well}\left(\mathrm{mBq}\right)}{\mathrm{number}\;\mathrm{of}\;\mathrm{cells}\;\times 100}$$

Efflux: Following incubation with 250 μL 0.2 MBq/mL [^99m^Tc]TcO_4_^−^ for 0.5 h, the medium was discarded, cells PBS-washed twice and incubated in 250 μL of radionuclide-free medium for up to 5 h. At indicated time points, the medium was collected, and a radioactive-free medium was added. At the last time point, the medium was collected, combined with two PBS washes and cells lysed in 1 M NaOH. Radioactivity was gamma-counted, and results expressed as percentage intracellular uptake of [^99m^Tc]TcO_4_^−^ with uptake at 0.5 h normalised to 100%.

### 4.4. Estimation of Subcellular [^99m^Tc]TcO_4_^−^ Distribution

Two methods were used; (i) subcellular fractionation [6,13] and (ii) microscopy-aided volume quantification of live cells and their nuclei. Fluorescence microscopy-aided volume determinations were performed for three possible scenarios; [^99m^Tc]TcO_4_^−^ located (i) only in the nucleus, or (ii) only in the extra-nuclear compartment, or (iii) homogenously distributed across the whole cell (Supplementary Methods and Figure S5).

### 4.5. Cytotoxicity Assays

After 0.5 or 24 h of incubation with [^99m^Tc]TcO_4_^−^ (0–4 MBq/mL; 250 µL) or EBRT at 0–8 Gy, culture supernatant was discarded, cells were washed twice with PBS and harvested with 0.25% trypsin containing 1 mM EDTA. Cells were seeded at 700–3000 into 6-well plates and cultured for 10–12 days. Colonies (≥50 cells) were fixed and stained for 20 min with 1% crystal violet in methanol (Sigma-Aldrich, Dorset, United Kingdom). The surviving fraction (SF) was determined using Equations (3,4):

(3) $$\mathrm{Plating}\;\mathrm{efficiency}\;\left(\mathrm{PE}\right)=\frac{\mathrm{number}\;\mathrm{of}\;\mathrm{colonies}\;\mathrm{formed}}{\mathrm{number}\;\mathrm{of}\;\mathrm{untreated}\;\mathrm{cells}\;\mathrm{seeded}}$$

(4) $$\mathrm{SF}=\frac{\mathrm{number}\;\mathrm{of}\;\mathrm{colonies}\;\mathrm{formed}\;\mathrm{post}\;\mathrm{treatment}}{\mathrm{number}\;\mathrm{of}\;\mathrm{cells}\;\mathrm{seeded}\;\times \;\mathrm{PE}\;}$$

### 4.6. Assessment of DNA Damage

Cellular assessment of DNA damage was quantified as double-strand breaks (DSB). Therefore, cells were treated as indicated and then fixed and stained with anti-phospho-histone H2AX (Ser139) antibody to detect DSBs in counter-stained nuclei using confocal fluorescence microscopy (for details, see Supplementary Methods).

### 4.7. Dosimetry

Dosimetry calculations were conducted using the MIRD formalism [26]. Briefly, the cell nucleus was chosen as the target, and the absorbed dose to the nucleus was estimated using self-dose. Live-cell confocal microscopy provided cell and nuclear radii of cells and nuclei (11 ± 2 and 6 ± 1 μm, respectively). Using MIRDcell V2.0 [26], S_self-dose_-values for different source volumes (cytoplasm and nucleus) were obtained to represent the absorbed dose rate in the target volume per unit activity inside each source volume (Table S1). The obtained S_self-dose_-values accounted for estimated cell and nuclei radii. Cumulative intracellular decays per cell in each source volume were estimated by calculating time-integrated activity per cell via trapezoidal integration using GraphPad Prism (v9.1.0), whereby uptake and efflux assay data served as input as well as data from subcellular fractionation and extra-nuclear/nucleus volumes. Following the MIRD formalism, [^99m^Tc]TcO_4_^−^ was assumed to accumulate uniformly across all cells, and the exponential growth of cells with time was taken into account, as well as the technetium-99m decay. Dose-response curves were fitted to the LQ-model using GraphPad Prism (v9.1.0).

### 4.8. Animal Tumour Model

Animal procedures were performed in accordance with UK legislation (PPL 70/8879) and approved by King’s College London AWERB. MDA-MB-231.hNIS-GFP xenografts were established by injecting 1 × 10^6^ cells into the left mammary fat pads of young adult female NSG mice. Twenty-eight days later, animals were randomly assigned to experimental groups (n = 2/cohort, 3 cohorts).

### 4.9. SPECT/CT Imaging

Twenty MBq or 200 MBq [^99m^Tc]TcO_4_^−^ in 200 µL were administrated intravenously under general anaesthesia (1.5–2.0% isoflurane in oxygen; 1.0–1.5 L/min). Half an hour post-administration, a 10 min CT-scan (1000 ms exposure time, 55 kVp, 1:4 binning, 180° rotation) was acquired, followed by a 45 min SPECT (20% energy window width (^99m^Tc; 112–168 keV), 40 projections and 60 s/frame) in helical scan mode using a nanoScan SPECT/CT (Mediso Ltd., Budapest, Hungary) with 4-head scanner with 9 × 1 mm diameter pinhole collimators. Data were reconstructed using the iterative reconstruction software Scivis HiSPECT v1.4.1876 (Scivis, Goettingen, Germany). Images were co-registered, processed, and analysed using VivoQuant v3.5-patch2 software (inviCRO, Massachusetts, USA).

### 4.10. Ex Vivo Analysis of Tissue Radioactivity

Relevant tissues were harvested and gamma-counted. Data were expressed as %IA/g tissue. Cohorts that had received 20 or 200 MBq radiotracers were first subjected to imaging, while the 125 MBq cohort was sacrificed 24 h after radiotracer administration.

### 4.11. Statistics

Three independent experiments were performed for all in vitro studies described, and data were represented as mean ± standard deviation. Graphs were plotted using GraphPad Prism v9.1.0. Statistical significance was calculated with either one-way ANOVA with Dunnett’s multiple comparisons test or two-way ANOVA with Tukey’s multiple comparisons test (5% significance level) unless stated otherwise.

## 5. Conclusions

This study in an epithelial breast cancer model informs on technetium-99m-induced in vitro DNA damage and reduction of cell survival. It reveals that tumour retention of [^99m^Tc]TcO_4_^−^ needed to be sufficiently long to cause damage if technetium-99m was internalised into cancer cells. Dosimetry suggested enhanced radiobiological effectiveness of technetium-99m when internalised into cells compared to EBRT. While further radiobiological investigations with different technetium-99m radiotracers are warranted, the values provided here will serve as a reference to develop future AE-therapy with technetium-99m developers and to assess the radiobiological risk from technetium-99m based imaging agents.

## Data Availability

The data presented in this study are available on request from the corresponding author.

## Supplementary Materials

The following are available online at https://www.mdpi.com/article/10.3390/ijms222413466/s1.
