# Supplementary material for: Relationship of In Vitro Toxicity of Technetium-99m to Subcellular Localisation and Absorbed Dose
Source: Int J Mol Sci. 2021 Dec 15;22(24):13466. doi: 10.3390/ijms222413466 (PMC8703725; doi:10.3390/ijms222413466)
Supplement: Supplementary file 1 [file ijms-22-13466-s001.zip › ijms-1492504-supplementary.pdf]

Supporting information for the article

Relationship of In Vitro Toxicity of Technetium-99m to Subcellular Localisation and Absorbed Dose

Ines M. Costa, Noor Siksek, Alessia Volpe, Francis Man, Katarzyna M. Osytek, Elise Verger, Giuseppe Schettino, Gilbert O. Fruhwirth and Samantha Y. A. Terry

This supplement consists of Supplementary Methods, one Supplementary Table, and five Supplementary Figures.

Supplementary Methods

Fluorescence-activated sorting

MDA-MB-231.hNIS-GFP cells were sorted by fluorescence-activated cell sorting (FACS) for GFP fluorescence. Cells were collected by centrifugation at 180× *g* for 5 min at room temperature and resuspended in phosphate buffered saline (PBS) containing 2% (v/v) FBS and 2.5 mM Ethylenediaminetetraacetic acid (EDTA) at a concentration of 20×10^6 cells/mL. Then, cell suspensions were collected in 5 mL round-bottom polystyrene tubes (Falcon™ 352235, FisherScientific). Using a FACSAriaTM III equipped with a 488 nm laser and a 100 µm nozzle, MDA-MB-231.hNIS-GFP cells within the sorting gate with the width set at 50% of peak height of GFP fluorescence intensity histogram were sorted and used for downstream cell culture and assays.

Flow cytometric quantification of hNIS-GFP expression over time

Every week either post-sorting cells or thawing respective stocks of sorted cells, MDA-MB-231.hNIS-GFP cells were resuspended in 2% (w/v) bovine serum albumin (BSA) and 2.5 mM EDTA in PBS at a concentration of 1×10^6 cells/mL. Cell suspensions were collected in 5 mL round-bottom polystyrene tubes (Falcon™ 352235, FisherScientific) and using a BD FACSMelody™ and settings for GFP (excitation 488 nm, emission 527/32), 20,000 single cell events were recorded per sample every week. Acquisition parameters were kept constant over time. Cells were discarded for experiments when MDA-MB-231.hNIS-GFP cells population was inferior to 95%.

Confocal microscopy of fixed MDA-MB-231.hNIS-GFP cells

2.0×10^5 MDA-MB-231.hNIS-GFP cells were seeded onto ethanol-cleaned 13 mm coverslips (VWR International Ltd, United Kingdom) in 24 well-plates incubated overnight at 37 °C in a humidified atmosphere with 5% CO_2. Medium was then removed, and cells were washed twice with PBS.

Coverslips were mounted onto microscope slices using ProLong Diamond Antifade mounting media formulated with blue DNA stain DAPI (ThermoFisher Scientific). Z-stack images (Z-step size: 0.4 μm) were acquired using the Nikon Eclipse Ti-E Inverted confocal microscope at Nikon Imaging Centre (King's College London) with an oil objective magnification of 60× and numerical aperture of 1.4 and appropriate settings for each fluorochrome (DAPI: excitation 405 nm, emission 450/50; GFP: excitation 488 nm, emission 525/50).

Estimation of subcellular [^{99m}Tc]TcO_4^- distribution

Two methods were used; (i) subcellular fractionation [6,13] and (ii) microscopy-aided volume quantification of live cells and their nuclei.

For subcellular fractionation studies, 2.5 × 10^6 cells were incubated with 250 μL 0.2 MBq/mL [^{99m}Tc]TcO_4^- in suspension for 0.5 h. Supernatants were collected, cells PBS-washed twice and cell pellets fractionated by using a kit as per manufacturer's instructions (#9038,Cell Signaling Technology). Radioactivity in samples was quantified by gamma-counting.

Fluorescence microscopy-aided volume determinations were performed for three possible scenarios; [^{99m}Tc]TcO_4^- located (i) only in the nucleus, or (ii) only in the extra-nuclear compartment, or (iii) homogenously distributed across the whole cell. To quantify whole cell and nuclear volumes, 2.5 × 10^4 cells were seeded onto 2-well chamber slides (Ibidi), allowed to adhere overnight and stained with Hoechst-33342 before imaging alive. Cells were kept in a chamber at 37 °C with 5% (v/v) CO_2 atmosphere and Z-stacks were acquired using an A1R Nikon Eclipse Ti-E Inverted confocal microscope. hNIS-GFP and Hoechst-33342 fluorescence enabled whole cell and nucleus volume determination from serial confocal Z-stacks.

Assessment of DNA damage

Cellular assessment of DNA damage was quantified as double strand breaks (DSB). MDA-MB-231.hNIS-GFP cells (2 × 10^5) were either exposed to EBRT (0–2 Gy) and incubated for 0.5 h or 24 h or treated with [^{99m}Tc]TcO_4^- (0–4 MBq/mL; 250 μL) for 0.5 h or 24 h. Indicated [^{99m}Tc]TcO_4^--treated samples were left to recover for another 24 h in radioactivity-free medium. At indicated time points, cells were fixed (4% formalin, 2% sucrose, PBS; 10 min), PBS-washed twice, permeabilised (0.8% Triton X-100, PBS, 10 min, RT) and PBS-washed twice before being blocked in 1% goat serum and 2% bovine serum albumin (BSA) in PBS for 2 h at RT. Samples were incubated with anti-phospho-histone H2AX (Ser139) mouse mAb (clone JBW301, Sigma-Aldrich; 1 μg/mL in 2% BSA/PBS; overnight at 4 °C). Subsequently, samples were rinsed twice with 2% BSA/PBS and stained with Cy5 AffiniPure goat anti-mouse IgG (H+L) (Jackson ImmunoResearch; 1 μg/mL in 2% BSA/PBS; 2 h at 4 °C in the dark). Samples were

mounted onto microscope slides with ProLong Diamond Antifade mounting media containing DAPI. Serial Z-stack images were acquired using a Nikon Eclipse A1 inverted confocal microscope (60x-NA1.4 oil objective). CellProfiler v3.1.9 was used to quantify γ-H2AX foci per nucleus.

Supplementary Table

Table S1. Summary of $S_{self\text{-}dose}$-values for spherical cells with a cell and nucleus radii of 11 ± 2 μm and 6 ±1 μm, respectively. $S_{self\text{-}dose}$-values were taken from MIRDcellv2 software [26].

Subcellular Localisation of $[^{99m}Tc]TcO_4^-$	Source Compartment	Target Volume	$S_{self\text{-}dose}$ (Gy/(Bq.s))
Nuclear fraction/compartment	Nucleus	Nucleus	4.96×10^{-4}
Extra-nuclear fraction/compartment	Cytoplasm	Nucleus	1.41×10^{-5}

Supplementary Figures

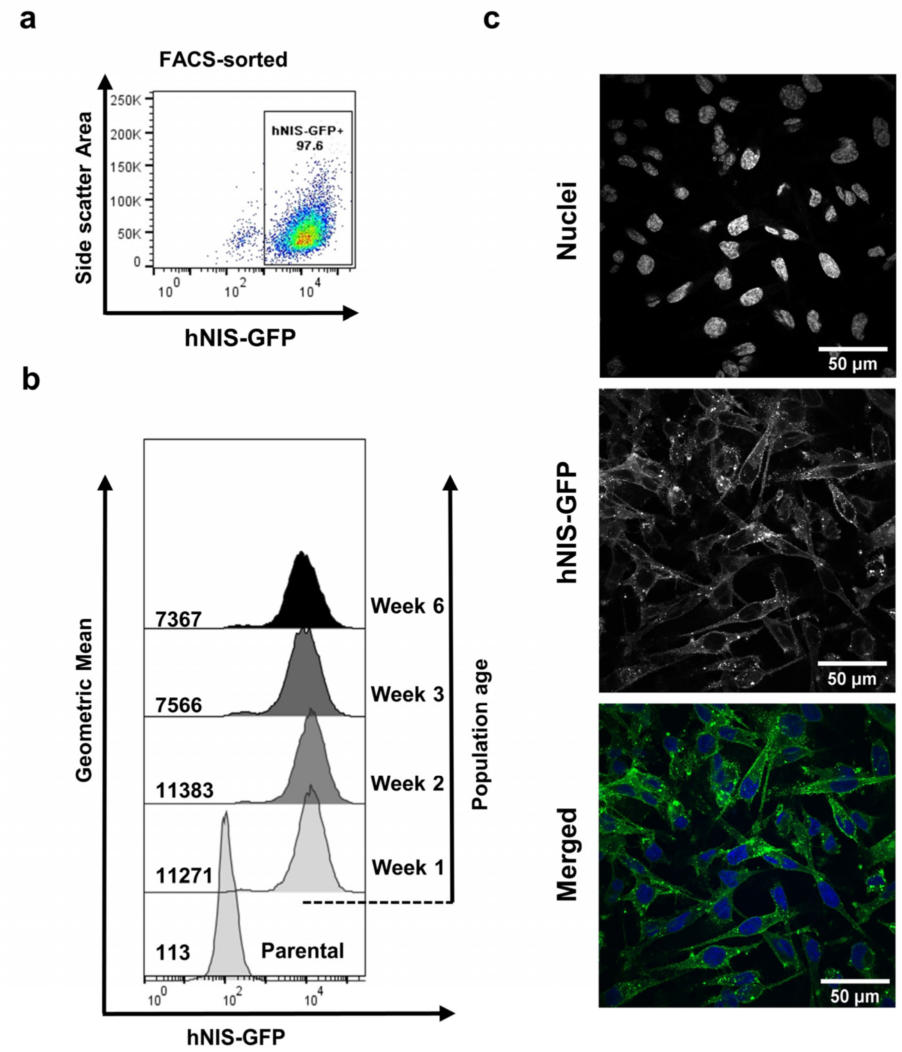

Figure S1. Expression and localization of hNIS-GFP in engineered MDA-MB-231.hNIS-GFP cells. (**a**) Flow cytometry reveals MDA-MB-231.hNIS-GFP cells to be ~98% pure and that (**b**) hNIS-GFP expression was stable over weeks. (**c**) Confocal microscopy demonstrated predominant plasma membrane localisation of hNIS-GFP.

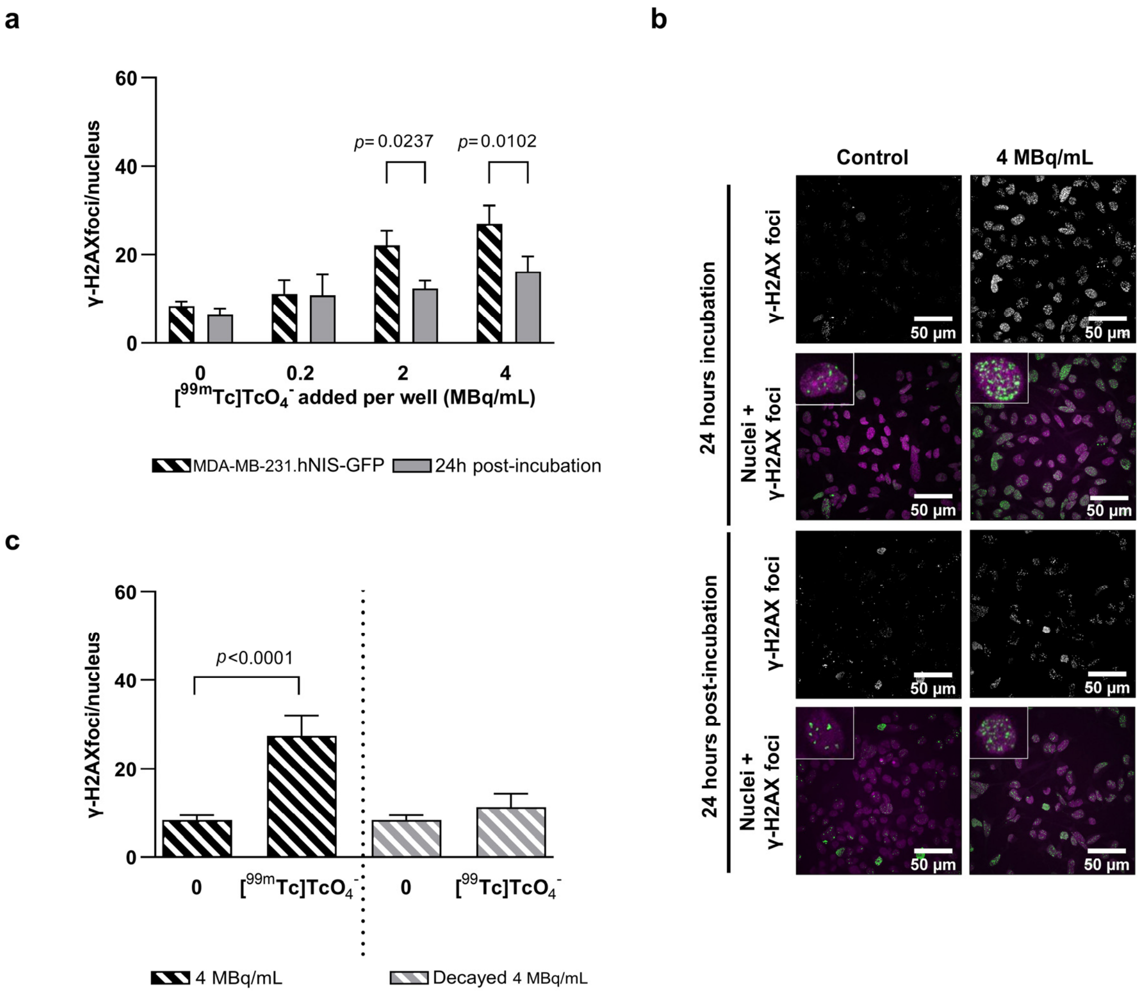

Figure S2. $[^{99m}Tc]TcO_4^-$ induced DNA damage. (**a**) Average number of γ-H2AX foci per nucleus following 24 h incubation with radioactive-free medium after 24 h incubation with increasing concentrations of $[^{99m}Tc]TcO_4^-$; (**b**) Representative confocal images of cells indicating γ-H2AX foci (green) within nuclei (magenta); (**c**) Average number of γ-H2AX foci per nucleus formation after 24 h incubation with 4 MBq/mL $[^{99m}Tc]TcO_4^-$ and decayed $[^{99}Tc]TcO_4^-$. Significant differences are indicated by brackets with corresponding p-values (2-way ANOVA with Tukey's multiple comparison correction except $[^{99}Tc]TcO_4^-$ vs untreated where Wilcoxon signed-rank t-test was used).

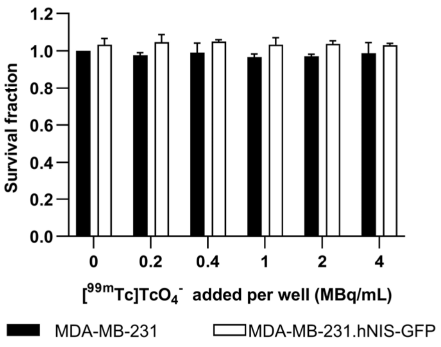

Figure S3. $[^{99m}Tc]TcO_4^-$ effect on clonogenic survival. Survival fraction of cells following 0.5 h incubation with increasing activity concentrations of $[^{99m}Tc]TcO_4^-$. Error bars represent SD and are centred around the means.

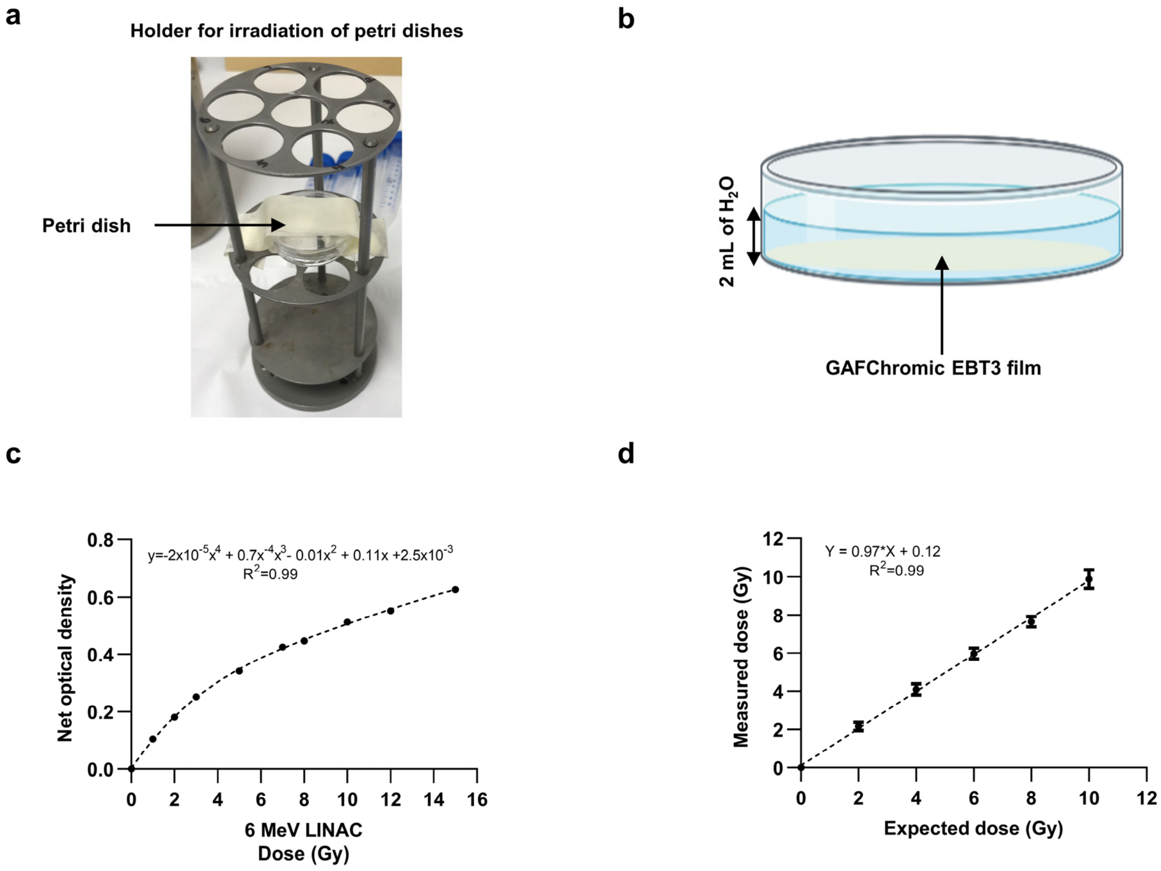

Figure S4. Control of irradiation of petri dishes with external beam irradiation therapy (EBRT). (**a**) Set-up of the petri dishes for irradiation. Petri-dishes were placed on the holder and tape was used to prevent movement during irradiation. (**b**) Representation of the set-up of petri dishes for irradiation. Gafchromic EBT3 films were placed at the bottom of petri dishes to mimic the irradiation of cells in a monolayer and 2 mL of H_2O were added to petri dishes to mimic the culture medium. (**c**) Calibration curve showing the net optical density as function of the irradiated films with increasing doses up to 15 Gy with a standard dose of high-energy X-rays (6 MeV) from a linear accelerator (LINAC). (**d**) Linear relationship between the expected dose absorbed by the films at the bottom of the petri dishes and the measured absorbed dose of films irradiated with increasing doses up to 10 Gy. Statistical significance was estimated using a paired t-test between the expected dose and measured dose. No significant difference between expected and measured dose was observed $p = 0.5874$. Data are represented as mean ± SD, n =3.

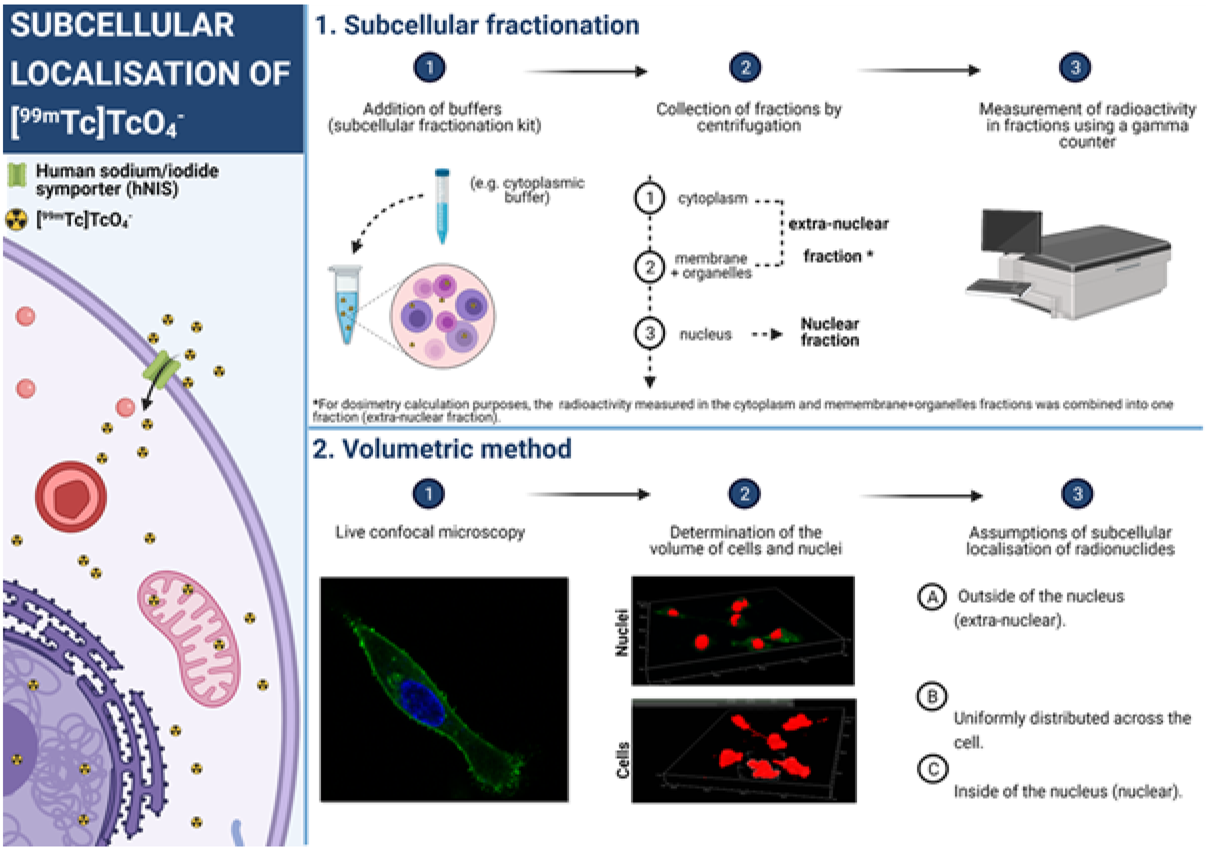

Figure S5. Schematic overview of methodology used to determine the subcellular localisation of [^{99m}Tc]TcO$_4^-$ in this study. Two methods were performed: (1) subcellular fractionation and (2) volumetric method.

Supplementary References

6. Maucksch, U.; Runge, R.; Wunderlich, G.; Freudenberg, R.; Naumann, A.; Kotzerke, J. Comparison of the radiotoxicity of the ^{99m}Tc-labeled compounds ^{99m}Tc-pertechnetate, ^{99m}Tc-HMPAO and ^{99m}Tc-MIBI. *Int. J. Radiat. Biol.* **2016**, *92*, 698–706, doi:10.3109/09553002.2016.1168533.

13. Costa, I.M.; Cheng, J.; Osytek, K.M.; Imberti, C.; Terry, S.Y.A. Methods and techniques for in vitro subcellular localization of radiopharmaceuticals and radionuclides. *Nucl. Med. Biol.* **2021**, *98–99*, 18–29, doi: 10.1016/j.nucmedbio.2021.03.010.0

26. Vaziri, B.; Wu, H.; Dhawan, A.P.; Du, P.; Howell, R.W. & SNMMI MIRD CommitteeMIRD pamphlet No. 25: MIRDcell V2.0 software tool for dosimetric analysis of biologic response of multicellular populations. *J. Nucl. Med.* **2014**, *55*, 1557–64, doi: 10.2967/jnumed.113.1310377

*** End of Supplement ***
